# Pandemics and Traditional Plant-Based Remedies. A Historical-Botanical Review in the Era of COVID19

**DOI:** 10.3389/fpls.2020.571042

**Published:** 2020-08-28

**Authors:** Sònia Garcia

**Affiliations:** Institut Botànic de Barcelona (IBB, CSIC-Ajuntament de Barcelona), Barcelona, Spain

**Keywords:** traditional medicine, plant-based alternatives, pandemics, antiviral, traditional knowledge

## Abstract

Pandemics are as old as humanity and since ancient times we have turned to plants to find solutions to health-related problems. Traditional medicines based mostly on plants are still the only therapeutic possibility in many developing countries, but even in the richest ones, herbal formulation currently receives increased attention. Plants are natural laboratories whose complex secondary metabolism produces a wealth of chemical compounds, leading to drug discovery – 25% of widespread use drugs are indeed of plant origin. Their therapeutic potential is even bigger: although many plant-based compounds show inhibitory effects against a myriad of pathogens, few reach the stage of clinical trials. Their mechanism of action is often unknown, yet traditional plant-based remedies have the advantage of a long-term experience in their use, usually of hundreds to thousands of years, and thus a precious experience on their safety and effects. Here I am providing a non-systematic historical-botanical review of some of the most devastating pandemics that humanity has faced, with a focus on plant therapeutic uses. I will revisit the Middle Ages black death, in which a plant-based lotion (the four thieves vinegar) showed some effectiveness; the smallpox, a viral disease that lead to the discovery of vaccination but for which the native Americans had a plant ally, an interesting carnivorous plant species; tuberculosis and the use of garlic; the Spanish flu and the widespread recommendation of eating onions, among other plant-based treatments; and malaria, whose first effective treatment, quinine, came from the bark of a Peruvian tree, properties already known by the Quechua people. Synthetic analogues of quinine such as chloroquine or hydroxychloroquine are now being revisited for the treatment of COVID19 symptoms, as they are artemisinin and derivatives, other plant-based compounds effective against malaria. Finally, I will give some hints on another facet of plants to aid us in the prevention of infectious diseases: the production of biotechnological plant-based vaccines. Altogether, my aim is to stress the significant role of plants in global health (past, present and future) and the need of enhancing and protecting the botanical knowledge, from systematics to conservation, from ecology to ethnobotany.

## Introduction

Pandemics have shaped the history of mankind, and plants were usually the first available therapeutic choice. There is evidence of herbal preparations by Egyptians around 1500 BC, later improved by Greeks and Romans, and widely documented in official drug books known as Pharmacopoeias. Still in our days, traditional medicines based mostly on plants are the only therapeutic possibility for many people in developing countries (Akerele, 1993). But, also in the first world, with wide access to the most modern drugs, the use of plant-based traditional medicine is experiencing a revival, as it is seen as safer and healthier than synthetic drugs. Indeed, one advantage of traditional remedies over modern drugs is that their effects and margin of safety have been known for long. There is also a renewed scientific interest on plant-derived drug discovery, according to the current increasing publication trend on the topic (Atanasov et al., 2015). The rich secondary metabolism that characterises plants make them a source of compounds that may have a yet unknown therapeutic potential, only limited by the availability of resources to perform clinical trials. It is claimed that natural products (mostly from plant origin) will be the most important source of new drugs in the future (Atanasov et al., 2015).

A recent editorial (Nature Plants, 2020) highlighted the need of funding and understanding botanical knowledge in the context of the current, and possibly future, pandemics. It is urgent to develop therapeutic tools to protect from high risk of infection (Mitjà and Clotet, 2020) and plant-based remedies with proven safety profiles could be one of the faster solutions. Here I present a non-systematic review with a historical-botanical perspective on some of the most important pandemics that humanity has faced, and in some cases is still facing, and how certain plants or plant-based remedies have been used, and may continue being used, to treat these diseases, possibly including COVID19.

## The Black Death

**Figure 1:**
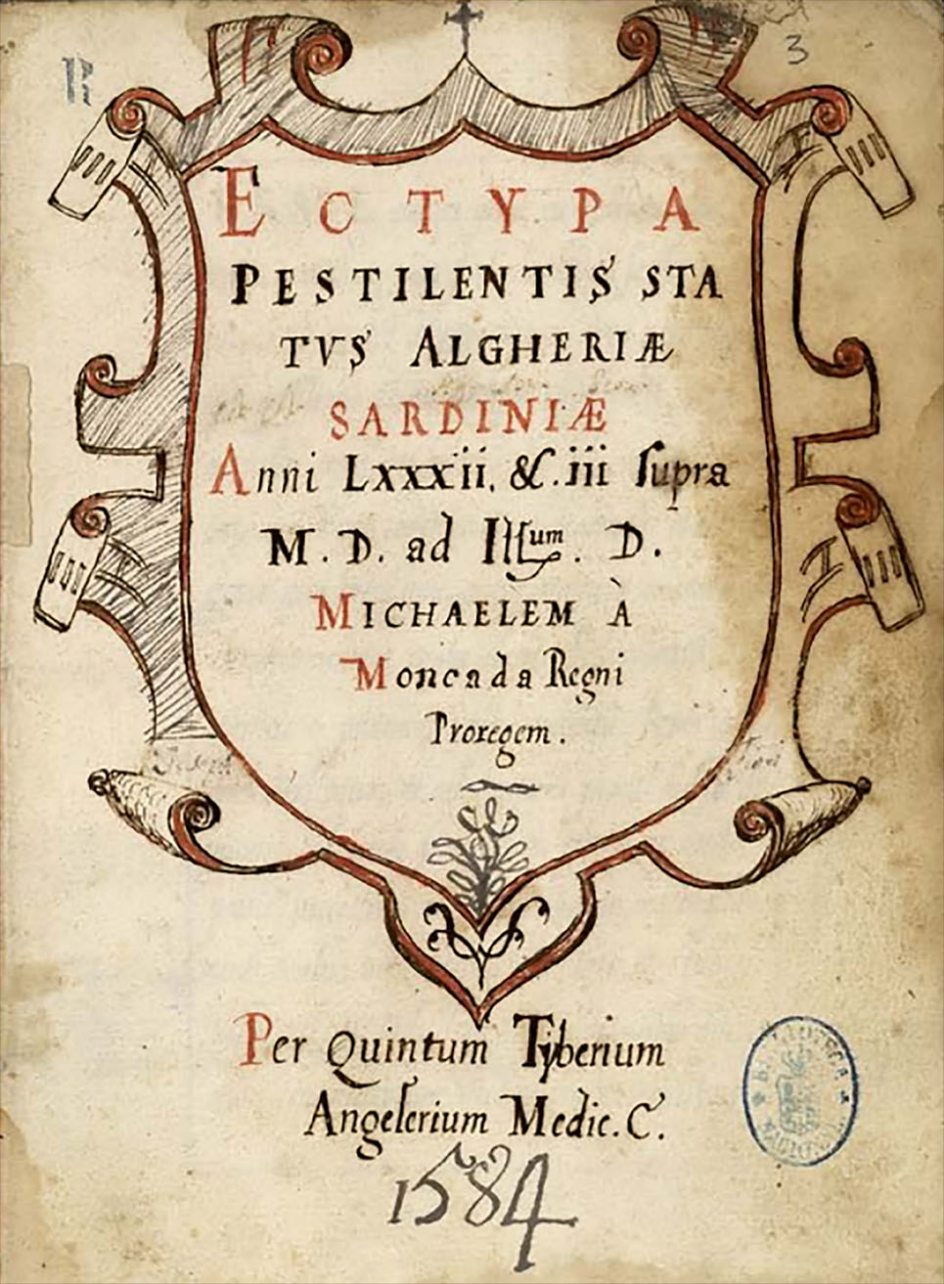
Cover of the manuscript “*Ectypa pestilentis status Algheriae Sardiniae anni LXXXII et III supra MD*” by Quintum Tyberio Angelerio, a physician in Alghero that faced the epidemy of black plague in 1582–1583 in Alghero (Sardinia). The text is written both in Latin and in Catalan. Image from the repository of the Biblioteca Nacional de España (BNE), under the CC-BY-NC-SA lnternational Creative Commons License.

The Black Death or Black Plague took place in the Middle Ages (1347–1351) in Eurasia, and still is the deadliest pandemic ever, with an estimated loss of 200 million of human lives wiping out 30 to 50% of European population in roughly four years (DeLeo and Hinnebusch, 2005). Although this is the most know outbreak of the bacterium *Yersinia pestis*, the much earlier -and longer- Plague of Justinian (from 541 to 750 AD) was also caused by the same pathogen (Harbeck et al., 2013), killing about 25–50 million people during two centuries. There have been other less spectacular, but still important plague outbreaks, arriving to the most recent ones in Madagascar during the present decade. Originated in China, the plague was usually spread by trade boats, whose rats carried fleas with the bacterium, which was transmitted to humans directly by the bite of the flea, and then between humans by contact or aerosol inhalation. There are several forms of the disease, the most common being the bubonic plague, which provokes the inflammation of the lymph nodes (buboes) as its most recognizable sign; a second form is the pneumonic plague which affects the respiratory system and is more deadly; the third form, the septicaemic plague, is the least common but has a mortality ca. 100% (Byrne, 2004). The antibiotic treatment, starting in early XXth century, reduced the death rate to about 1%–5% which previously was between 40%–60%; however, little is known on the remedies used before antibiotics were a reality and the major plague outbreaks occurred much earlier. In the Middle Ages, some preventive measures included, among others, carrying sweet smelling herbs to clear “the evil air” (which was believed to carry the pathogen) around the person (Jones, 2000), garlic for cleaning kidneys and liver, and lavender or chamomile teas to calm the stomach bile (Khaytin, 2019). A remedy named “the four thieves vinegar” was very popular: it consisted in several herbs, such as angelica (*Angelica archangelica*), camphor (*Cinnamomum camphora*), cloves (*Syzygium aromaticum*), garlic (*Allium sativum*), marjoram (*Origanum majorana*), meadowsweet (*Filipendula ulmaria*), wormwood (*Artemisia absinthium*), and sage (*Salvia officinalis*), brewed in vinegar (Gattefosse, 1937). Before going out, people should apply it on hands and face for avoiding to contract the plague. Some of these plants are well known flea repellents, so this may be one of the reasons for its efficacy. Other herbs such as meadowsweet might have been included to release pain (as it contains salicylic acid, a precursor of aspirin) and to mask odours, a very helpful property considering that decomposing bodies were usually encountered – the legend states that the name of the remedy might refer to thieves using it to rob the plague dead or sick (Lucas, 1969). Another treatment coming from ancient Greeks also gained popularity: the King Mithridates antidote (Totelin, 2004), an extract of about fifty plants in a mixture with opium (*Papaver somniferum*) paste, which if any, at least eased the pain or promoted a peaceful death. Other prescriptions included lavender or rosewater baths, probably due to their antimicrobial and buboes healing properties. Willow bark (another source of salicylates) was also given as a painkiller (Khaytin, 2019). A curious “prophylactic” plant-based remedy was recommended by the Napolitan doctor Angelerio during a plague outbreak in Alguero in the XVI century (1582–1583) (**Figure 1**): “any person going out from home must carry a cane (note: probably from the species *Arundo donax*) six spans long, and as long as the cane is, one must not approach other people” (Bianucci et al., 2013).

## Smallpox

The origin of smallpox, a viral infectious disease caused by Variola virus (two variants: *V. major* and *V. minor*) is unknown, but it dates back at least to the ancient Egypt (third century BC), since some mummies showed smallpox-like eruption, the characteristic macules of the disease. As with the plague and other pandemics, the disease has occurred in several outbreaks around the world, the most recent in the late 60s. This virus has killed between 300 and 500 million people during the 20^th^ century (Koplow, 2003) until the global eradication campaign by the World Health Organisation (WHO) in 1967. Smallpox was the first infectious disease to have been eradicated (1980), the (only) second one being rinderpest, a viral illness of cattle. The smallpox vaccine (the first ever) was based on Edward Jenner’s demonstration, by the end of the XVIII^th^ century, that inoculation with cowpox (a variant of the smallpox virus infecting cows) protected against the disease. Actually, Jenner’s contribution popularized the practice of vaccination, a word coined by himself coming from the latin word *vaccinus* (i.e., or/from the cow) for the prevention of several other infectious diseases.

**Figure 2:**
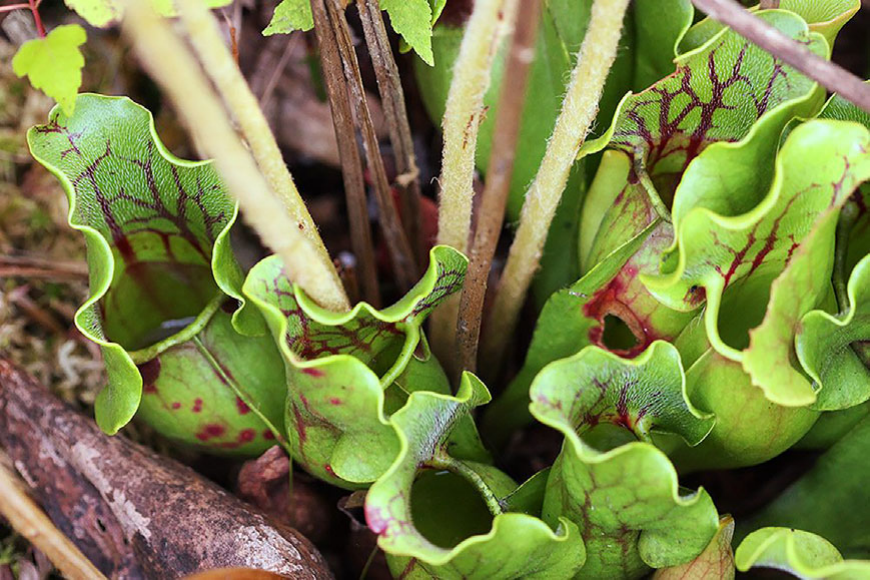
The carnivorous purple pitcher plant, *Sarracenia purpurea*, a folk remedy by the Native Americans to treat smallpox. The image shows the “pitchers” which are traps to little invertebrates. Image taken at the John Bryan State Park (Ohio, USA) courtesy of Elizabeth McGee from Curious Plant (https://curiousplant.com/carnivorous-plants-ohio/).

However, before vaccination was discovered, how did people deal with the illness? Particularly interesting was the approach of the Native Americans, which were deeply affected by the disease. By the end of XIX^th^ century several surgeons and practitioners related to the US army, as well as the prestigious botanist Charles F. Millspaugh (1892), described the use of poultices and infusions from the Indigenous medical flora based on the plant *Sarracenia purpurea* (family Sarraceniaceae) to be effective for treating smallpox, in a likely case of medical appropriation of the Indigenous therapeutic knowledge (Lawrence-Mackey, 2019). Known by Native Americans (Mi’kmaq people) as Mqo’oqewi’k, also named purple pitcher plant, it belongs to a genus of carnivorous species that use modified pitcher-shaped leaves to trap insects. Possibly, the spotted appearance of the plant (**Figure 2**), resembling one of the main clinical signs of the disease (Clarke, 1996), inspired its use to the Indigenous people. This may be another example of the doctrine of signatures, an ancient concept by which God somehow indicated to men what plants would be useful for, by certain signs (Coles, 1657), a pseudoscience which has caused more harm than good in general, although exceptions appear. Compelling descriptions of their effectiveness were recorded, such as ‘‘the greatest remedy known for the dreadful scourge’’ or “‘it seemed to arrest the development of the pustules, killing, as it were, the virus from within” (Clarke, 1996). The advent of vaccination put forward the botanical remedy, but the antiviral properties of *Sarracenia purpurea* have been later demonstrated *in vitro* (Arndt et al., 2012). The authors showed that the plant extract was not only active against smallpox, but also against other poxviruses, papovirus SV-40 and various herpes viruses, including papillomavirus and Epstein-Barr virus-associated carcinomas, usually by inhibiting the virus replication at the level of early transcription (Moore and Langland, 2018).

## Tuberculosis

Another global and persistent pandemic is tuberculosis, caused by *Mycobacterium tuberculosis*. Together with smallpox, it is one of the oldest known diseases, since molecular data and archaeological evidence support that it coexisted with humans from the Neolithic (Gutierrez et al., 2005; Nicklisch et al., 2012). Relevant figures in the history of medicine such as Hippocrates or Avicenna identified the disease, which involved coughing blood and fever and which was often lethal. Actually, Avicenna detected the infectious nature of the illness and based on tuberculosis, was probably the first to come up with the idea of quarantine to stop the spread of infectious diseases (Shephard, 2015). Tuberculosis infected 10 million people only in 2019, of which 1.5 million died (UNAIDS, 2020). It is found in every country, being the first infectious cause of death worldwide. From the HIV/AIDS outbreak, the combination of both is usually fatal where tuberculosis is endemic (mainly developing countries), as the immune weakening caused by HIV facilitates the onset of tuberculosis. This disease is indeed the final cause of death of many HIV infected people; paradoxically, while many could live now with AIDS they are dying from tuberculosis. The main reason is that the bacterium has developed resistance to most antibiotics, which usually have to be taken during long and tedious treatments. This is why researchers have turned to the search of effective alternatives also among medicinal plants, as some of them have already demonstrated anti-tuberculosis activity. Besides, an effective plant-based treatment would be more affordable in poor countries which are those more affected by the disease. Among the plants that are being investigated, garlic (*Allium sativum*, family Alliaceae), a former remedy already used to treat the plague (see above) stands out for its renowned properties, although it still far from being an alternative. The benefits of garlic (of which there are about 300 varieties) are well-known already from the Ayurvedic and Unani medicine systems (Raghunandana et al., 1946) as well as from the Chinese traditional medicine, but also ancient Greeks, Romans and even Egyptians used it to treat illnesses. It has been used as a food and folk medicine for centuries by many cultures. Garlic has a variety of pharmacological virtues, including antimicrobial, anticancer, antioxidant (Dini et al., 2011), fungicidal and as a cure for heart diseases, among others (Majewski, 2014). The antitubercular and other antimicrobial activities of garlic, however, have been demonstrated *in vitro* but still, seldom *in vivo*. Although garlic has more than 2000 biologically active compounds, allicin is the most relevant, albeit highly unstable; therefore, depending on the preparation of the garlic-based remedy the efficacy may not be as high as expected (Majewski, 2014). The wide antimicrobial and even antifungal spectrum of allicin is explained by its inhibitory effects on sulfhydryl metabolic enzymes. By interacting with these enzymes, allicin induces thiol stress in bacteria, which, among others, inhibits the growth of the microorganisms (Müller et al., 2016).

## Malaria

Malaria, caused by protozoan species of the *Plasmodium* group, is an infectious disease coming from the bite of a mosquito, usually an infected *Anopheles* sp. female. The current name of the illness was given by the Italians around XIX^th^ century, as a contraction of the words “mal aria” (i.e., bad air) from the belief that the disease was transmitted by the “miasma” coming from marshes (Macip, 2020). The most typical symptom is fever, together with nausea and vomiting, tiredness, headache, occasionally yellow skin and in severe cases it can lead to seizures, coma and death. It is another ancient disease, spanning from the Neolithic to our days, and currently found in all intertropical continents. Recent studies detected the parasite in African monkeys, probably being the source of the disease, although it is still debated how it spread worldwide (Molina-Cruz and Barillas-Mury, 2014). It is known that malaria arrived to Europe by the first century AD, probably coming from the African rainforests and travelling by the Nile to the Mediterranean, where it spread to the Middle East and from there to Greece, Italy - historians hypothesize on the triggering role of malaria in the fall of the Roman Empire - and the rest of Europe, even as far as England and Denmark (Karlen, 1996). Between the XVI^th^ - XIX^th^ centuries the disease crossed the Atlantic Ocean probably on slave ships to reach the American continent (Yalcindag et al., 2012). It was suffered by presidents of North America such as G. Washington or A. Lincoln and it raged specially with Native Americans, taking thousands of their lives. The Centers for Disease Control and Prevention, the leading national public health institute of the United States, was founded because of malaria in 1946. In the last century, probably 150–300 million people have died from the disease, accounting for 2%–5% of deaths (Carter and Mendis, 2002). At present malaria is most-worrying in sub-Saharan Africa, accounting for ca. 90% of current cases, although there is also a resurge in southern Asia (Arrow, 2004).

The most well-known and one of the most effective historical treatments against malaria is quinine, an alkaloid extracted from the bark of the cinchona tree (*Cinchona officinalis*) belonging to family Rubiaceae, the same of coffee. It is original from Peru (where it is the national tree, although currently it is considered an endangered species) and the Quechua traditionally used the ground bark of these trees to stop shivering because of cold, not for malaria treatment *per se*. Most likely, Spanish Jesuits missionaries brought cinchona to Europe for the first time, having observed how the Quechuas used it to threat shivering, by the end of XVI^th^ century - a second case of medical appropriation in this story. The tree was named (by C. Linnaeus) after the Spanish Countess of Chinchon, who was treated with its bark in Peru back in the early XVI^th^ – Linneaus misspelled the name of the countess, omitting the first “h” in the name (Meshnick, 1998). It is also said that the Countess may have introduced the curative bark to Europe when she returned to Spain, but it is currently considered that this a legend rather than what actually happened (Haggis, 1941). Quinine is effective on the “cessation of febrile paroxysms” (Stephens et al., 1917), one of the main symptoms of malaria, and which has given its popular name to the species (fever tree). Malaria outbreaks, however, continued to appear during centuries with no alternative to quinine. During World War I the German army was strongly affected by the parasite in the troops of East Africa, as the Allies controlled Java, the main worldwide quinine producer. In an interesting historical moment that impelled science, the German government commissioned a search for a substitute to quinine, determined not to suffer again from its shortage. In 1934 chloroquine, a synthetic compound similar to quinine was synthesized at the Bayer laboratories. Much later (1955) another very similar derivative, hydroxychloroquine, was produced in the US. Both chloroquine and hydroxychloroquine are used to prevent and treat malaria, being some of the antimalarial drugs of choice in areas where the disease is not resistant to them (a recurrent problem in this disease); they were preferred over quinine because of much less severe adverse effects, although at present there are many other even safer alternative drugs for the treatment of malaria. In recent years, these antimalarials have shown several immunomodulatory effects and they currently treat, mostly, diseases such as lupus erythematosus or rheumatoid arthritis.

**Figure 3:**
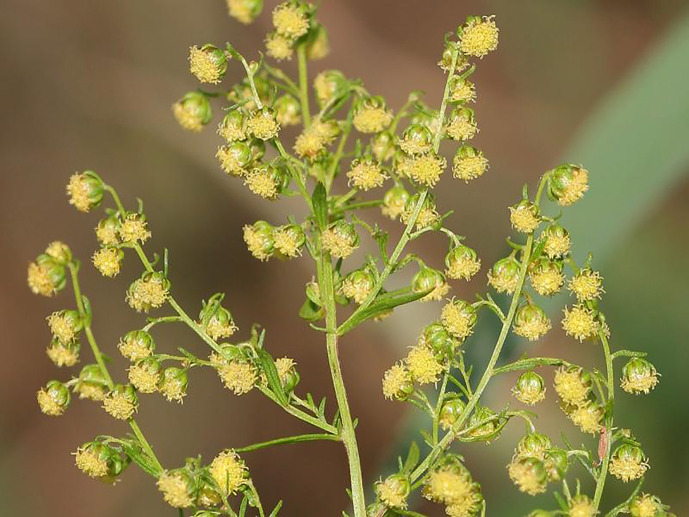
Synflorescences of the sweet wormwood, *Artemisia annua*. Image by Kristian Peters, under the CC BY-SA 3.0 International Creative Commons License.

As with tuberculosis, the parasite tended to develop resistance to these treatments sooner or later, and researchers were urged to look for alternatives. A very popular one was found in the plant *Artemisia annua* (sweet wormwood, from family Asteraceae) (**Figure 3**) a remedy known in Chinese traditional medicine as qing-hao for more than 2000 years (Klayman, 1985). Chinese herbalists had been using it for treating haemorrhoids, chills, and fevers (Trigg and Kondrachine, 1998). The species, as other members of genus *Artemisia* such as absinthe or tarragon, is aromatic and bitter. One of the compounds responsible for its bitterness, a sesquiterpene lactone extracted from the glandular trichomes named artemisinin, is the active compound against malaria. The discovery of artemisinin is also very remarkable from the historical point of view. In 1967, during the Chinese Cultural Revolution under Mao Zedong’s mandate, the secret “Project 523” was a plant screening research program to find an alternative treatment for malaria, which was ravaging Vietnamese army during Vietnam war. In 1972, Dr. Youyou Tu a researcher of that program, isolated artemisinin, “rediscovering” the ancient remedy qing-hao. The drug started to be used in 1979, a relatively short-period to establish a new medicine in the market, but it was also based on thousands of years of experience by the Chinese traditional practitioners. Nowadays artemisinin and its synthetic derivatives are one of the main defences against drug-resistant malaria in the Asiatic southeast. However, WHO recommends it in combined therapy with other drugs, in part to avoid the development of resistance and in part to counteract the short half-life of artemisinin in plasma, leading to the Artemisinin Combination Therapies (ACTs) which include companion drugs such as some cloroquine derivatives (e.g., mefloquine). In 2015 Tu was awarded the Nobel Prize for the discovery of artemisinin, which represents an important contribution of China to the global health, as well as the first and awaited Nobel prize in the sciences for China. It is considered the most significant milestone of tropical medicine of the last century, contributing to a better health and saving tens of thousands of lives every year in tropical developing countries of South Asia, South America and Africa.

## Spanish Flu

**Figure 4:**
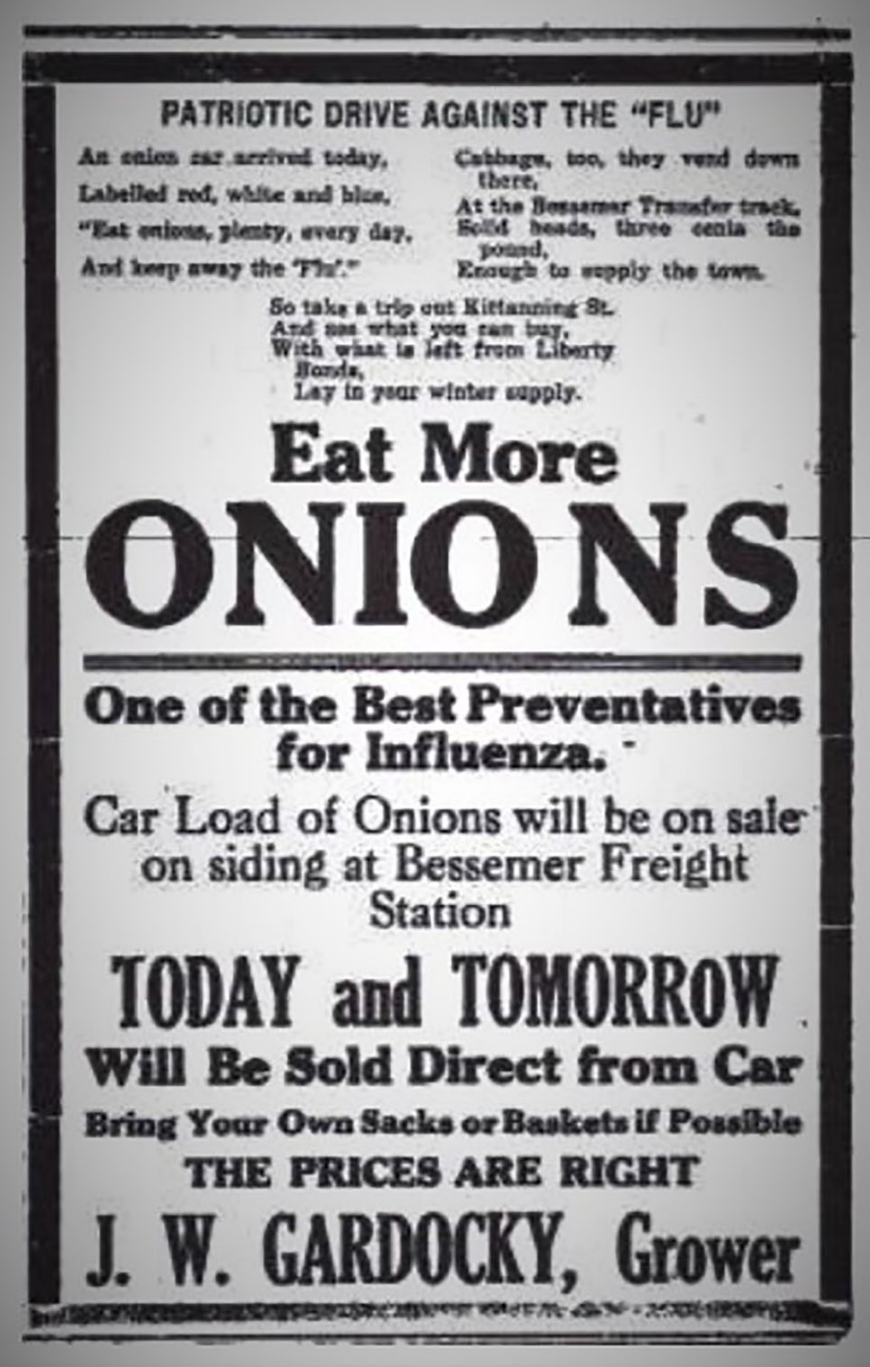
Propaganda poster that got popular during the 1918 flu pandemic in the USA. From reddit.com, image under the CC-BY-NC-SA lnternational Creative Commons License.

In the spring of 1918 started one of the deadliest pandemics in recent history, caused by one aggressive strain of the H1N1 influenza virus (the same virus that caused the 2009 swine flu pandemic). It was popularly known as the Spanish flu because in the context of World War I censorship minimized the effects of the pandemic to keep people’s morale in the countries involved in the conflict, while in the neutral Spain newspapers were free to report its effects, giving the impression that this country had been particularly devastated by the disease. During 15 months, until summer 1919, there were three waves of the pandemic, being the second the worst. A fourth, much fainter wave, took place in the spring of 1920 and after this one, the virus disappeared as it had arrived. It infected about half a billion people (ca. 1/3 of the world’s population) and killed about 50 million (with some estimates as high as 100 million); for a reference World War I estimates range from 15 to 22 million deaths. The origin of the virus is unclear but it is thought that it started as a zoonosis from birds to humans which later was transmitted from humans to swine. The symptoms were an amplified version of those of normal flu, but typically deaths were caused by complications derived from a secondary pneumonia. Contrary to other H1N1 flu strains, this one was unusually lethal among young people, and almost one century later its high-virulence is only partly understood (Tumpey et al., 2005). Since at that time there was neither vaccine, nor antibiotics to treat secondary pneumonia, the main prophylactic options were, as with the COVID19 pandemic, to avoid contact through lockdown and quarantines, to increase personal hygiene and to use disinfectants widely. Also, people turned to folk remedies and some recommendations got popular, such as the widespread advice “Eat more onions!” (**Figure 4**) (Arnold, 2018). As with garlic, onion (*Allium cepa*) has certain compounds (particularly a polyphenol named quercetin) which have demonstrated antiviral properties (Lee et al., 2012; Sharma, 2019) but still more research and clinical trials are needed. Besides, in the USA a group of doctors known as “The Eclectics” got positive results by treating the flu symptoms with plant remedies, together with other measures that included exercise. They reported a fatality rate ca. 0.6% for their patients while the average in that pandemic was ca. 3% (Abascal, 2006). By selecting the herbs to match the symptoms, they used a wide variety of species. The most remarkable among them, and that have later proved therapeutic, were: *Gelsemium sempervirens* (known as yellow yasmine, with antipyretic properties), *Eupatorium perfoliatum* (boneset, already known by native Americans to treat cold-related symptoms), *Actea racemosa* (black cohosh, also used by Native Americans as a painkiller, probably due to the content in salicylic acids of its roots) and *Asclepias tuberosa* (pleurisy root, used to treat respiratory problems and with expectorating properties) (Abascal, 2006). However, despite their long history of use, again there is little applied research on these plants. Currently, flu is partly under control by the release of annual vaccination campaigns with newly synthesized vaccines that collect most of the virus’ seasonal variability. In the latter most important flu pandemic (2009) besides the vaccine, oseltamivir (Tamiflu^®^) a drug derived from the species *Ilicium verum* (star anise, from family Schisandraceae) was also crucial to treat most severe cases, although the production of this compound is limited by the low productivity of the tree, and synthetic derivatives are being developed (Macip, 2020). Finding adequate treatments for flu is still and urgent task as the fear of a pandemic similar to the one in 1918 is a still a sword of Damocles in the concerns of most epidemiologists.

## The Possibility of Plant-Based Vaccines

From the 1980s, the science of “molecular farming” gives another potential role to plants on the prevention of infectious diseases, involving plants or plant cell cultures to produce recombinant proteins (Rybicki, 2014). The first steps of this approach were the “manufacturing” of the human growth hormone, monoclonal antibodies or human serum albumin in transgenic tobacco or sunflower plants (Barta et al., 1986; Hiatt et al., 1989; Sijmons et al., 1990). Other recombinant proteins more recently produced in plants -shifted from bacterial, mammalian or fungal cell to plants and plant cell cultures- and commercialized, include human type collagen I manufactured in tobacco, bovine trypsin in maize or human lysozyme and lactoferrin in rice (Yang et al., 2003; Hennegan et al., 2005; Shoseyov et al., 2014; Takeyama et al., 2015). As with the mentioned proteins, the vaccine production would follow similar steps: isolation of a specific antigen protein, the one that triggers an immune response from the virus; the gene(s) encoding that protein is transferred to bacteria and these bacteria are used to infect plants, so the plant will in turn produce the antigen protein – the vaccine. Plants would provide a flexible, cheap and easily scalable method to manufacture vaccines. They would also be safer than traditional vaccines, because of the absence of pathogens of animal origin. Plant-based vaccines for humans are not yet in the market, although some candidates have entered clinical trials (Rosales-Mendoza et al., 2020). It is likely, however, that they will start being approved in the mid-term, at least in the cases of Ebola or rabies, or in a longer term for seasonal influenza (Rybicki, 2014).

## What Plant-Based and Plant-Related Treatments Can Offer to COVID19 Therapy?

The outbreak of COVID19 caused by the coronavirus SARS-CoV-2, originated in China (province of Wuhan) in December 2019 and has caused 7,238,484 infections and 409,644 deaths worldwide (updated 9^th^ June 2020). Even earlier than the pandemic status was declared by WHO (11^th^ March 2020) researchers across the world engaged in hundreds of clinical trials, in an unprecedented quest for a cure to the disease, vaccine, drug or both. Given the long time-frames that usually imply finding a good candidate, many research groups have turned to repurpose other drugs. The reasons are that the effects (including adverse or side effects) of these drugs are well known and have been used in broad population groups with different ages and idiosyncrasies, so the security margin is increased, allowing to save precious time in long trials. In this regard, antimalarials are potential candidates (Schlagenhauf et al., 2020) and both chloroquine but particularly hydroxychloroquine (as explained above, synthetic derivatives of quinine, the antimalarial alkaloid coming from the bark of the fever tree) are being studied to fight COVID19, although it is perhaps too soon to draw conclusions on their efficacy. Several studies have reported their utility for some patients and some national guidelines have recommended both drugs for treatment of COVID19 (see Colson et al., 2020 and Singh et al., 2020) despite there has been certain controversy. The WHO halted studies on these drugs by the end of May 2020 prompted by an observational study reporting that hydroxychloroquine produced a higher mortality rate in hospitalised patients, but the study was soon retracted on the basis of questionable veracity of data and analysis (Mehra et al., 2020) and trials on the drug have been resumed shortly after. The effectiveness of hydroxycloroquine taken at initial stages of the disease was recently tested in a multicentre randomised controlled trial based on previous experiences of Post Exposure Prophylaxis (PEP) drugs to prevent infections (Mitjà and Clotet, 2020), but no benefit was observed beyond the usual care (Mitjà et al., 2020). Other plant-based antimalarials, artemisinin and derivatives, are also being tested against SARS-CoV-2, again not without controversy. In many African countries, an elixir based on *Artemisia annua* extract, “covid-organics” is being distributed as a cure against COVID19. However, there is little scientific evidence of the effectiveness of such elixir and its extended consumption can have associated problems, the most important the development of resistance to the drug by the malaria parasites in a continent particularly sensitive to the disease. Nevertheless, there is evidence that the extract of *Artemisia annua* has antiviral properties, being active against SARS-CoV-1 (Li et al., 2005), herpes simplex (Karamoddini et al., 2011), hepatitis A (Seo et al., 2017), hepatitis B, bovine viral diarrhoea, and Epstein-Barr (Haq et al., 2020). This has stimulated the research of the potential use of artemisinin and derivatives (such as artesunate) to treat COVID19, which is now being conducted by several biotech companies (e.g., Mateon Therapeutics, ArtemiLife) as well as by public research institutions (e.g., the Liverpool School of Tropical Medicine, the Max Planck Institute of Colloids and Interfaces). Traditional Chinese Medicine has also had a say in the cure of COVID19: the National Administration of Traditional Chinese Medicine (NATCM) organized a study in late January 2020 to identify potential treatments, and the lung cleansing and detoxifying decoction (LCDD) was widely used and studied through clinical trials; its apparently high effectiveness made that the NATCM officially recommended LCDD as a treatment for COVID 19 (Weng, 2020). Among the LCDD 21 ingredients, there were species such as *Ephedra sinica* (well known as decongestant and bronchodilator through the active compound ephedrine), *Atractylodes macrocephala* (showing antiviral activity against influenza viruses in experimental assays) or *Scutellaria baicalensis* (containing anti-inflammatory flavonoids), and the combination of these effects and others from the remaining ingredients likely counteracts COVID19 by their synergistic activities. However, as Weng (2020) points out, it is difficult to transfer the success of the LCDD treatment to other countries, both because the cultural acceptance of TCM is not present outsides China, and due to the lack of knowledge on the precise chemical composition and mechanism of action, which are required in modern therapy.

Once the first vaccine for COVID19 is finally developed (with estimates ranging from September 2020 to several years) another plant may also play an important role in order to produce it in large amounts: *Nicotiana benthamiana*, a close relative to tobacco. This species is the focus of the EU project NEWCOTIANA, coordinated by researcher Dr. Diego Orzáez at the CSIC, the leading public research body in Spain. In this project, genome editing practises (e.g., CRISPR) will be used to transform the plant in a biofactory for the large-scale production of the vaccine once it is available. Moreover, one company may be currently developing a COVID19 vaccine based on the expression of a SARS-CoV-2 protein in tobacco - Kentuchy Bioprocessing, a biotechnological branch of British American Tobacco (Capell et al., 2020). As Rosales-Mendoza et al. (2020) stated in a recent review, the production of a plant-based vaccine in the context of the current pandemic would have the advantages of low cost, fast and escalable production, easy administration, and safety.

Beyond plant-based vaccines, molecular farming through plants, usually by transient expression of target proteins, can also be deployed to produce diagnostic reagents, as well as antibodies and antiviral proteins for therapeutic use. The Italian biotechnology company Diamante is generating antigens, to use in ELISA tests (serological), based on a SARS-CoV-2 protein also in tobacco plants (Capell et al., 2020). Another EU consortium, Pharma-Factory is also developing plant-based platforms to produce medical, veterinary and diagnostic products, for dealing with COVID19 and also other diseases.

## Conclusion

I hope that the reader finds this review useful to call for the important role that plants have played and still play in human health. As Schlagenhauf et al. (2020) commented, plant-based remedies are, more than an “alternative medicine”, the organisms to which we owe some of the most useful therapeutic tools. Still in the era of wide implementation of (synthetic) drug treatments, we turn to plants in many cases when resistances appear, as shown. Paradoxically, there is a human tendency to ignore plants, a form of cognitive bias known as “plant blindness” (Allen, 2003) that should be opposed, perhaps by enhancing and implementing more widely the botanical education. In this context, it is also essential not only to maintain but to increase societal funding into basic sciences such as botany, as well as to foster collaboration between scientists from different disciplines, whose interaction may open new therapeutic possibilities. Finally, I would like that this review serves as a little recognition to the usually ignored ethnobotanical traditional knowledge of many indigenous peoples across the world of which the so-called Western culture has in most occasions, illegitimately appropriated.

## Author Contributions

The author confirms being the sole contributor of this work and has approved it for publication.

## Funding

This work was supported by the Spanish [CGL2016-75694-P (AEI/FEDER, UE)] and Catalan [grant number 2017SGR1116] governments. SG is the holder of a Ramón y Cajal contract (RYC-2014-16608).

## Conflict of Interest

The author declares that the research was conducted in the absence of any commercial or financial relationships that could be construed as a potential conflict of interest.
